# Origins, diversity, and adaptive evolution of DWV in the honey bees of the Azores: the impact of the invasive mite *Varroa destructor*

**DOI:** 10.1093/ve/veae053

**Published:** 2024-07-15

**Authors:** Ana R Lopes, Matthew Low, Raquel Martín-Hernández, M Alice Pinto, Joachim R De Miranda

**Affiliations:** Centro de Investigação de Montanha (CIMO), Instituto Politécnico de Bragança, Campus de Santa Apolónia, Bragança 5300-253, Portugal; Laboratório Associado para a Sustentabilidade e Tecnologia em Regiões de Montanha (SusTEC), Instituto Politécnico de Bragança, Campus de Santa Apolónia, Bragança 5300-253, Portugal; REQUIMTE-LAQV, Faculdade de Farmácia, Universidade do Porto, Rua de Jorge Viterbo Ferreira, 228, Porto 4050-313, Portugal; Department of Ecology, Swedish University of Agricultural Sciences, Uppsala 756-51, Sweden; Centro de Investigación Apícola y Agroambiental (CIAPA), IRIAF. Instituto Regional de Investigación y Desarrollo Agroalimentario y Forestal, Marchamalo 19180, Spain; Instituto de Recursos Humanos para la Ciencia y la Tecnología (INCRECYT-FEDER), Fundación Parque Científico y Tecnológico de Castilla—La Mancha, Albacete 02006, Spain; Centro de Investigação de Montanha (CIMO), Instituto Politécnico de Bragança, Campus de Santa Apolónia, Bragança 5300-253, Portugal; Laboratório Associado para a Sustentabilidade e Tecnologia em Regiões de Montanha (SusTEC), Instituto Politécnico de Bragança, Campus de Santa Apolónia, Bragança 5300-253, Portugal; Department of Ecology, Swedish University of Agricultural Sciences, Uppsala 756-51, Sweden

**Keywords:** *Apis mellifera*, *Varroa destructor*, deformed wing virus, Azores archipelago, ASV genetic diversity, virus quasispecies, invasive vector

## Abstract

Deformed wing virus (DWV) is a honey bee virus, whose emergence from relative obscurity is driven by the recent host-switch, adaptation, and global dispersal of the ectoparasitic mite *Varroa destructor* (a highly efficient vector of DWV) to reproduction on honey bees (*Apis mellifera*). Our study examines how varroa affects the continuing evolution of DWV, using the Azores archipelago, where varroa is present on only three out of the eight Islands, as a natural experimental system for comparing different evolutionary conditions and trajectories. We combined qPCR of 494 honey bee colonies sampled across the archipelago with amplicon deep sequencing to reveal how the DWV genetic landscape is altered by varroa. Two of the varroa-free Islands were also free of DWV, while a further two Islands were intriguingly dominated by the rare DWV-C major variant. The other four Islands, including the three varroa-infested Islands, were dominated by the common DWV-A and DWV-B variants. The varroa-infested Islands had, as expected, an elevated DWV prevalence relative to the uninfested Islands, but not elevated DWV loads, due the relatively high prevalence and loads of DWV-C on the varroa-free Islands. This establishes the Azores as a stable refuge for DWV-C and provides the most convincing evidence to date that at least some major strains of DWV may be capable of not just surviving, but actually thriving in honey bees in the absence of varroa-mediated transmission. We did not detect any change in DWV genetic diversity associated with island varroa status but did find a positive association of DWV diversity with virus load, irrespective of island varroa status.

## Introduction

Although many factors, such as climate change, pesticides and altered agricultural and beekeeping practices, have contributed to increasing honey bee mortality worldwide (Hristov et al. 2021), the single most significant driver of colony losses continues to be *Varroa destructor*, primarily through the lethal virus epidemics it transmits (Yañez et al. 2020b). The critical moment in this development was the mass introduction of the Western honey bee *Apis mellifera* to Southeast Asia during the Green Revolution of the 1950s–1960s (Traynor et al. 2020). This brought *A. mellifera* in close contact with the Eastern honey bee (*Apis cerana*) and its coevolved obligate ectoparasitic mite *Varroa jacobsoni*. This mite readily adapted to the new host and evolved into a distinct species, *Varroa destructor* (hereafter “varroa”), with *A. mellifera* serving as its primary host (Anderson and Trueman 2000, Roberts et al. 2015). Varroa subsequently rapidly dispersed throughout the *A. mellifera* populations of the world during the 1970s–1990s (Boncristiani et al. 2021), strongly facilitated by the largely unregulated global trade in honey bees (Traynor et al. 2020). While *A. cerana* has evolved a series of physiological and behavioral adaptations that naturally limit varroa population growth, ensuring their stable coexistence (Grindrod and Martin 2023), these adaptations are far less developed or lacking in *A. mellifera* (Traynor et al. 2020, Gabel et al. 2023, Grindrod and Martin 2023), resulting in an unstable coexistence that can only be mitigated by beekeeping practices to restrict varroa population development, generally through the application of chemical acaricides (Traynor et al. 2020).

The varroa life cycle is relatively simple and is divided into two phases: a phoretic phase, when adult mites feed and disperse on adult honey bees (Han et al. 2024); and a reproductive phase, when female mites invade honey bee prepupal cells to feed and reproduce on developing pupae (Traynor et al. 2020, Han et al. 2024). This reproductive phase is by far the most damaging to the host, both for individual bees and for the colony as a whole, and this damage is almost entirely due to the viruses that are transmitted by varroa during feeding (Yañez et al. 2020b). Since it is these vectored viruses, rather than the mite per se, that are responsible for most of the varroa-associated bee mortality, it is also through the evolution of these virus assemblages that much of the adaptation of varroa to a coexistence with *A. mellifera*, and with beekeeping, occurs.

The evolutionary ecology of honey bee viruses in relation to varroa occurs at three trophic levels. The first, and most direct, is at the individual bee level, particularly the pupal phase, where virus ecological succession and virulence evolution are driven forward by the semi-exponential replication of competing virus species, strains, and variants, whose overall virulence is restricted by the absolute requirement for the pupa to survive to emergence, in order to release the reproducing mites for vectoring and ensure the survival of the virus assemblage (Mondet et al. 2016). Virus evolution at this level is highly efficient and relatively straightforward, and driven mostly by the molecular, cellular, and physiological conditions of the host and by virus population genetic dynamics, as formulated by the “quasispecies” theoretical framework (Dolan et al. 2018, Gisder et al. 2018, Ryabov et al. 2019, Yañez et al. 2020a). Evolution at this level can be influenced by the honey bees themselves through the differential detection and removal of moribund varroa-infested pupae, thus removing excessively virulent virus assemblages from the colony (Mondet et al. 2016).

The second trophic level is the colony, where virus proliferation and evolution are driven forward by the growth of the bee and mite populations through the foraging and brood-rearing activities and high turnover of the adult worker bee population, in response to external and internal environmental cues, and whose overall virulence is restricted by the absolute requirement for the colony to survive long broodless periods with a sufficient mass of healthy worker bees to restart brood-rearing with a new influx of fresh pollen. Virus evolution at this level is complex and relatively inefficient, driven mostly by within-colony epidemiological criteria involving multiple alternative transmission routes for different viruses and strains (Yañez et al. 2020b), and by mostly sublethal effects on adult bees, brood, and varroa. Evolution at this level can be influenced by beekeeping practices, particularly varroa treatment, supplemental feeding and the culling (or saving) of weak colonies, possibly leading to contrasting effects on the virulence evolution of the virus assemblages (Neumann et al. 2012, Brosi et al. 2017, Traynor et al. 2020, Ray et al. 2023).

The third trophic level is geographic, where virus evolution and ecological succession are driven forward by the introduction of varroa into previously varroa-free areas, and is restricted by natural or artificial (e.g. quarantine) barriers to such introductions, as well as the natural range limits for *A. mellifera* survival imposed by climate. Evolution at this level can be influenced by (migratory) beekeeping, the seasonal transport of colonies into and out of particular geographic areas, and by climate change, which can shift the natural range of plants and the corresponding foraging criteria for honey bees.

Out of this multi-level cauldron of evolutionary, epidemiological, and ecological pressure, Deformed wing virus (DWV; *Iflavirus aladeformis*) has emerged from relative obscurity (De Miranda and Genersch 2010, Mondet et al. 2014, Martin and Brettell 2019) as the virus most optimally adapted to varroa-mediated transmission in *Apis mellifera*, and in beekeeping. As a result, DWV currently occupies a dominant position in the honey bee virus ecosystem (Martin and Brettell 2019, Beaurepaire et al. 2020, Traynor et al. 2020), including through spill-over to susceptible local non-*Apis* bees, ants, and wasps (Yañez et al. 2020b). The abundance and virulence of DWV are directly linked to the mode of transmission (varroa), rather than an innate property of the virus itself, which in the absence of varroa reverts towards obscurity (Doublet et al. 2024, Lopes et al. 2024). The innately avirulent character of DWV is likely due to its strongly developed sexual and vertical transmission route (Fievet et al. 2006, Yañez et al. 2020b), where avirulence is a positive adaptive trait and any virus-induced reduction in host fitness is severely punished, particularly for the haploid drones through their long sexual maturation (approximately half the adult drone life span) and extremely strenuous mating flight (Yañez et al. 2012). This is where modern honey bee breeding, involving artificial queen rearing and insemination, could influence DWV evolution by bypassing these natural selection mechanisms against DWV virulence evolution (Brosi et al. 2017).

The adaptation of DWV to varroa, honey bees, and beekeeping is an ongoing, dynamic process following orthodox virus epidemiological and adaptive evolutionary patterns, with the consecutive emergence of increasingly better-adapted major strains displacing previously dominant variants, including the possible extinction of obsolete strains. Through this process, we currently know of four major DWV variants (DWV-A, -B, -C, and -D; in order of discovery: Ongus et al. (2004); Lanzi et al. (2006); Mordecai et al. (2016); De Miranda et al. (2022)), of which DWV-B is currently the dominant strain worldwide, displacing the previously dominant DWV-A strain (Grindrod et al. 2021, Paxton et al. 2022), with DWV-C being exceedingly rare and DWV-D suspected to be extinct (De Miranda et al. 2022). This pattern also roughly matches the phylogeographic reconstruction of the genetic evolution of DWV in *V. destructor* and *A. mellifera*, with DWV-A thought to be the original strain that dispersed worldwide with varroa during the initial expansion phase and with DWV-B emerging several decades later and gradually displacing DWV-A during the last decade (Grindrod et al. 2021, Paxton et al. 2022, Hasegawa et al. 2023).

Since the time of Darwin, isolated island archipelagoes have been supreme natural laboratories for studying how adaptive ecological and evolutionary processes interact to shape the diversity patterns of populations (Whittaker and Fernández-Palacios 2007) due to their natural boundaries and geographical isolation within a relatively constant overall environment, creating well-defined and relatively similar replicate units to aid statistical analysis. Their small size, separation, and geographic isolation also mean that they are often last to be affected by global-scale invasions, thus becoming historical refuges and time capsules for unique populations. In this study, we took advantage of the spatially and temporally heterogeneous distribution of varroa across the mid-Atlantic Azores archipelago, combined with a clear historical record of honey bee movement to and between the Islands, to investigate how varroa invasion has impacted the epidemiology, genetic diversity, and evolution of DWV viral communities in Azorean honey bee populations. In doing so, we discovered several important novel insights into the adaptive relationships among honey bees, varroa, and the major DWV variants; into the possible biogeographic origins of DWV; into the primary drivers shaping DWV genetic diversity; and into how DWV genetic diversity could be used to confirm, or reconstruct, historical invasion scenarios.

## Results

### Deformed wing virus prevalence, but not Deformed wing virus load, is related to island varroa invasion status

**Figure 1.**
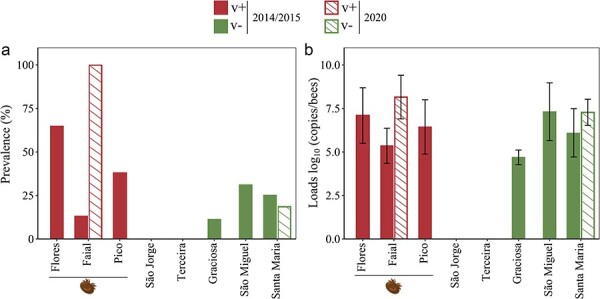
DWV prevalence (A) and loads (B) as determined using the qPCR assay designed for the *RdRp* region, on the islands with (v+, red) and without (v-, green) varroa, sampled in 2014/2015 (solid bars) and 2020 (light bars). All 145 colonies sampled from the islands São Jorge (43 colonies) and Terceira (102 colonies) tested negative for DWV, both those sampled in 2014/2015 (87 colonies) and in 2020 (58 colonies). Mean viral loads ± standard deviation (error bars). The varroa icon was obtained from www.biorender.com under a post-doc license.

DWV was detected on six of the eight Islands, including the three Islands with varroa (v+: Flores, Faial, and Pico) and three of the Islands without varroa (v-: Graciosa, São Miguel, and Santa Maria). All 145 colonies sampled from the v- Islands of São Jorge (43 colonies) and Terceira (102 colonies) tested negative for DWV, both of which were sampled in 2014/2015 and in 2020 (Fig. 1a). The average DWV prevalence in the Azores was 22% (110/494 colonies), although it was greater on the v+ Islands (13.0–64.9%) than on the v- Islands (11.1–31.0%). When we subjected these data to Bayesian inference modeling, we found strong evidence (100% probability) that the presence of varroa on an island was related to higher DWV prevalence, increasing from a mean prevalence estimate of ∼5% on the v- Islands to 27% on the v+ Islands (mean increase in prevalence = 22.2 ± 8.6%; Table 1). When we excluded the Islands São Jorge and Terceira (both without DWV) from the analyses, we still obtained a strongly elevated mean DWV prevalence on the v+ Islands compared to the v- Islands (98.2% probability).

**Table 1.** DWV prevalence, log_10_-loads, richness, evenness, and Shannon-Wiener index estimates for honey bee colonies on islands where varroa has been detected (v+) *versus* islands where varroa has not been detected (v-) in the Azores. These estimates are the mean ± SD of the posterior distributions generated from Bayesian hierarchical models that account for apiary and year effects for DWV. Also presented is the mean expected difference in prevalence, log-loads and diversity measures for colonies on v+ islands compared to v- islands (Effect size v+), and the probability that this difference results in a higher prevalence, log-loads, and diversity on v+ islands (Pr (pos > neg)).

Response variables	Estimates			
	Islands v-	Islands v+	Effect size v+	Pr (pos > neg)
Prevalence	4.8 ± 1.9%	27.0 ± 9.1%	22.2 ± 8.6%	100%
Log_10_-DWV loads	7.20 ± 0.34	7.19 ± 0.34	−0.01 ± 0.37	48%
ASV Richness	8.436 ± 1.149	9.915 ± 1.158	1.478 ± 1.621	84%
ASV Evenness	0.248 ± 0.039	0.250 ± 0.035	0.002 ± 0.032	53%
Shannon-Wiener Index	0.640 ± 0.100	0.654 ± 0.092	0.014 ± 0.081	58%

In the comprehensive 2014/2015 sampling, the mean viral loads in the colonies on the v+ and v- Islands were similar, ranging from 5.4 ± 1.0 log_10_ copies/bee (Faial) to 7.1 ± 1.6 log_10_ copies/bee (Flores) on the v+ Islands, compared to 4.7 ± 0.4 log_10_ copies/bee (Graciosa) to 7.3 ± 1.6 log_10_ copies/bee (São Miguel) on the v- Islands (Fig. 1b; Supplementary Table S1). This overall similarity in DWV loads between Islands with and without varroa persisted in the more limited 2020 resampling, but with higher average loads on both Islands where DWV was detected, from 6.1 to 7.3 log_10_ copies/bee on Santa Maria (v- island) and from 5.4 to 8.2 log_10_ copies/bee on Faial (v+ island). The other Islands included in the 2020 resampling (São Jorge and Terceira) remained DWV free between 2014/2015 and 2020. When we implemented Bayesian modeling, we did not find statistical support for the effect of varroa invasion status on DWV loads (Pr = 48%, Table 1), which was not surprising given the high loads found on colonies from the easternmost v- Islands of São Miguel and Santa Maria (Supplementary Table S1). These results indicate that, in this study, the presence of varroa clearly increased DWV prevalence, but did not contribute to higher DWV loads in DWV-positive colonies. Since these findings run counter to everything that is currently known about the relationship between varroa and DWV (De Miranda and Genersch 2010, Martin et al. 2012, Mondet et al. 2014, Martin and Brettell 2019, Doublet et al. 2024), we closely examined the genetic makeup of the DWV populations on these Islands.

### The Deformed wing virus genetic landscape is altered by varroa invasion

The DWV genetic diversity in the Azores was represented by 366 unique amplicon sequence variants (ASVs) for the amplified fragment. DWV-B contributed the most unique ASVs (155), followed by DWV-A (133) and DWV-C (78). No ASV was recovered that uniquely matched DWV-D (De Miranda et al. 2022). Most of the ASVs (212) were only detected in a single colony each, with only a handful of ASVs truly widely distributed among all the colonies (Supplementary Fig. S1). In each individual sample, the single most abundant (“dominant”) ASV was identified, as well as the second and third most abundant ASVs (Supplementary Table S1; Supplementary Fig. S2). This produced a set of 20 different dominant ASVs across all the 102 DWV-infected samples of which 9 ASVs were “super-dominant” (i.e. dominant in more than one colony), with the remaining 11 ASVs dominant in only a single colony each (Supplementary Table S1). Interestingly, while DWV-B provided the most unique ASVs (155), it provided the fewest dominant and super-dominant ASVs (2). By contrast, DWV-C provided the most dominant ASVs (10) even though it had the fewest total ASVs (78), while DWV-A provided the most super-dominant ASVs (4). The nine super-dominant ASVs also frequently appear as either the second or third most common ASV, in those colonies where they are not the first most dominant ASV (Table 2A). There is also a progressively greater diversity among the second and third most common ASVs as we go deeper into the quasispecies of each sample (Supplementary Table S1). In almost all samples, the most dominant ASV occupied >40% of the quasi-species, while the second ASV occupied >20% of the quasispecies in only about a quarter of samples, with the third (fourth, fifth, etc.) ASVs occupy progressively smaller fractions of the quasispecies, in fewer of the samples (Table 2B; Supplementary Fig. S2).

**Table 2.** Patterns of ASV dominance and superdominance among the 102 DWV-positive samples. (A) Tabulation of the identity and clade of the most common 1^st^, 2^nd^ and 3^rd^ most abundant ASVs, ranked by the frequency of the most common superdominant ASV. ‘Singletons’ refers all those instances combined where a particular ASV only is (super)dominant in a single colony. The clade of each ASV is indicated in brackets (B) Number and percentage of colonies where the proportion of reads ascribed to the 1^st^, 2^nd^, 3^rd^, 4^th^ and 5^th^ most abundant ASV exceeds the indicated percentage.

**A**	**ASV dominance & superdominance**					
ASV superdominance	1st ASV		2nd ASV	3rd ASV		
1st superdominant ASV	ASV10 (DWV-A)		ASV23 (DWV-B)	ASV3 (DWV-C)		
2nd superdominant ASV	ASV3 (DWV-C)		ASV24 (DWV-B)	ASV24 (DWV-B)		
3rd superdominant ASV	ASV1 (DWV-A)		ASV3 (DWV-C)	ASV23 (DWV-B)		
4th superdominant ASV	ASV24 (DWV-B)		ASV10 (DWV-A)	ASV10 (DWV-A)		
5th superdominant ASV	ASV13 (DWV-A)		ASV13 (DWV-A)	ASV13 (DWV-A)		
6th superdominant ASV	ASV16 (DWV-C)		ASV1 (DWV-A)	ASV1 (DWV-A)		
7th superdominant ASV	ASV39 (DWV-C)		ASV39 (DWV-C)	ASV43 (DWV-A)		
8th superdominant ASV	ASV23 (DWV-B)		ASV50 (DWV-A)	ASV80 (DWV-A)		
9th superdominant ASV	ASV33 (DWV-A)		ASV173 (DWV-C)	ASV114 (DWV-C)		
10th superdominant ASV	Singletons		ASV80 (DWV-A)	ASV30 (DWV-C)		
11th superdominant ASV	Singletons		Singletons	ASV144 (DWV-B)		
12th superdominant ASV	Singletons		Singletons	ASV36 (DWV-B)		
13th superdominant ASV	Singletons		Singletons	ASV106 (DWV-B)		
14th superdominant ASV	Singletons		Singletons	ASV165 (DWV-A)		
15th superdominant ASV	Singletons		Singletons	Singletons		
**B**	**DWV quasispecies ASV composition**					
		Proportion ASV in sample				
Number of colonies		>10%	>20%	>30%	>40%	
1st ASV		102	102	101	95	
2nd ASV		47	27	15	3	
3rd ASV		14	4	0	0	
4th ASV		3	0			
5th ASV		0				
		Proportion ASV in sample				
Percentage of colonies		>10%	>20%	>30%	>40%	
1st ASV		100.0	100.0	99.0	93.1	
2nd ASV		46.1	26.5	14.7	2.9	
3rd ASV		13.7	3.9	0.0	0.0	
4th ASV		2.9	0.0			
5th ASV		0.0				

**Figure 2.**
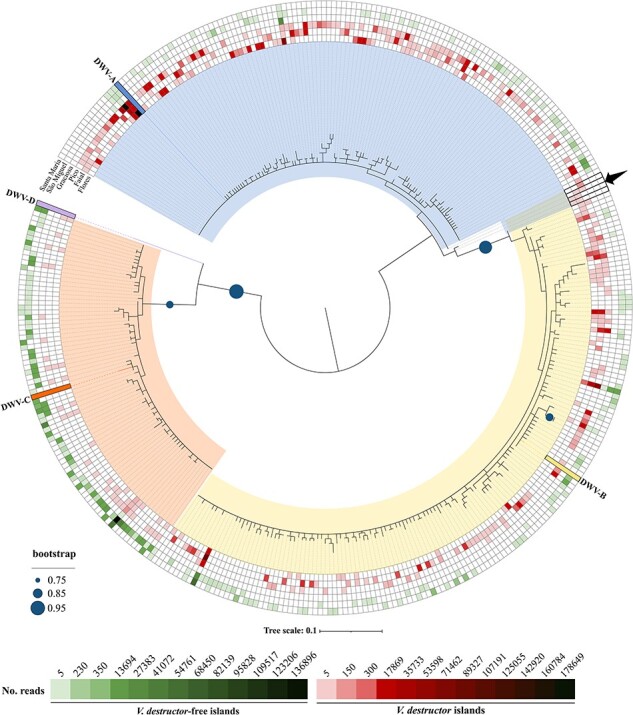
Phylogenetic tree of the *RdRp* region constructed from the 366 ASVs identified in the 102 DWV-positive colony samples. DWV-master references from GenBank are marked in bold (DWV-A: Acc. number AY292384; DWV-B: Acc. number AY251269; DWV-C: Acc. number ERS657949; DWV-D: Acc. number MT504363) and different colours were used to represent each master variant: blue for DWV-A, yellow for DWV-B, orange for DWV-C, and violet for DWV-D. The evolutionary history was inferred from the maximum likelihood method using the Tamura 3-parameter model (bootstrap = 1000 replicates). The heatmap surrounding the phylogenetic tree was conducted in iTOL online tool (https://itol.embl.de) using the number of reads for each ASV per island as input file. The green gradients denote the islands without varroa and the red gradients the islands with varroa. The four sequences between the DWV-A (blue) and DWV-B (yellow) clades, marked with an arrow, are recombinants between DWV-A and DWV-B.

**Figure 3.**
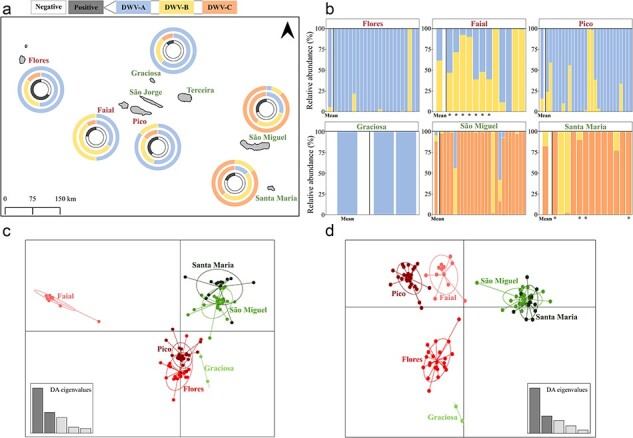
Patterns of DWV master variants in the Azores. (a) Geographic structure and proportion of DWV master variants across the eight islands. The outer ring represents the number of ASVs for each of the master variants when only considering the 20 single most abundant ASVs identified in each of the 102 DWV-positive colony samples. The middle ring represents the number of ASVs for each of the master variants when considering all 366 unique ASVs, andthe inner ring represents the proportion of DWV-positive (black) and DWV-negative (white) samples. (b) Relative abundance of DWV master variants by island and by colony, using the 366 unique ASVs. The first bar shows the mean relative abundance for the whole island. Asterisks (*) represent colonies sampled in 2020. (c) Discriminant Analysis of Principal Components (DAPC) for the 102 DWV-positive colony samples when using all 366 unique ASVs found across all islands and colonies. (d) DAPC when using only the single most abundant ASV found in each of the 102 DWV-positive colony samples (20 ASVs total). The varroa-infested islands (Pico, Faial and Flores) are represented by shades of red, the varroa-free islands (Graciosa São Miguel and Santa Maria) by shades of green. The Azores map in 3A was constructed with QGis 3.22 (www.qgis.org) using a base map downloaded from DIVA-GIS (www.diva-gis.org), both used with permission.

**Figure 4.**
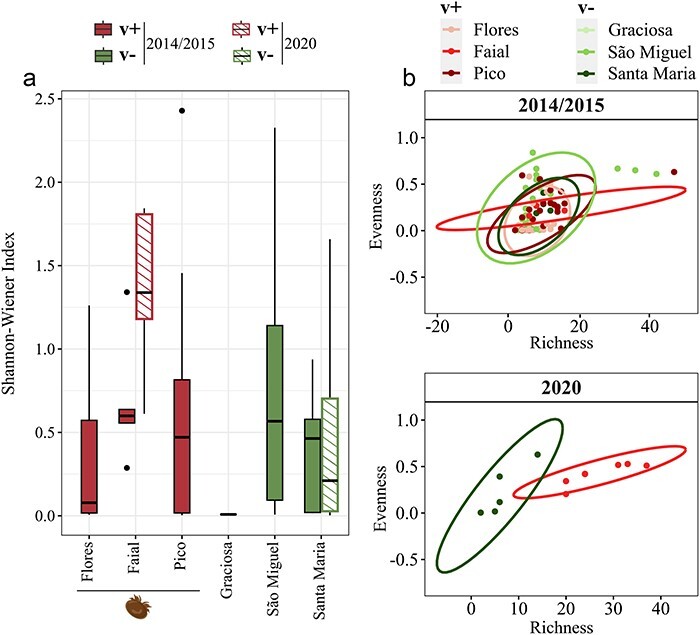
Alpha diversity of DWV samples by island and varroa status for the 2014/2015 and 2020 sampling periods. (a) Shannon-Wiener index. (b) Evenness-richness plots, with ellipses indicating a confidence level of 95%. Red scales were allocated to varroa (v+) islands, while green scales were allocated to varroa-free (v-) islands. The varroa icon was obtained from www.biorender.com under a post-doc license.

The phylogenetic relationship between the 366 ASVs and their distribution on the DWV-positive Islands with and without varroa is shown in Fig. 2. The ASVs separated into three large clades corresponding to the three main DWV master strains: DWV-A, DWV-B, and DWV-C. Within each clade, the topology is flat and dominated by short, poorly supported terminal branches, with little evidence of lineal descent among the ASVs. Four ASVs were slightly separated from the main DWV-A and DWV-B clades. One of these (ASV-324) is essentially DWV-A with several unique Single Nucleotide Polymorphisms (SNPs) across the fragment. Two of these (ASV-113 and ASV-293) are identical DWV-ABA recombinants distinguished by a single SNP, and the fourth (AVS-291) is a DWV-BA recombinant with a couple of unique SNPs. Notably, no recombinant ASV involving DWV-C and either DWV-A or DWV-B was detected, despite all three master strains co-occurring in 73.5% of the colonies (Supplementary Table S2). The recombinant ASVs were only detected on the v+ Islands. Since we were able to recover 4 recombinant ASVs among the 366 ASVs, despite focusing on a very short fragment (210 bp) that is furthermore not located in known recombination hotspots on the DWV genome (Moore et al. 2011, Wang et al. 2013, Ryabov et al. 2014, Dalmon et al. 2017), it is highly likely that more recombination events between these strains exist elsewhere along the genome. In the second part of Fig. 2, the three green and three red outer rings visualize the global quantitative distribution of these 366 ASVs on the six Islands, as determined from the sequencing read counts. The main observation from these rings was that all three master strains (DWV-A, DWV-B, and DWV-C) were found on all six DWV-infected Islands and, in fact, co-occurred in the vast majority of individual DWV-infected colonies across all Islands (73.5%; Supplementary Table S2). A second observation from these global distribution rings was that the DWV-A ASVs were far more dominant on the v+ Islands than on the v- Islands, while the reverse was true for the DWV-C ASVs, with the DWV-B ASVs somewhere in between (Fig. 2). However, there was great variation in how the ASVs from the different master clades were distributed in the individual colonies on these Islands (Fig. 3a and b; Supplementary Fig. S2). A third key observation is that for each of the three DWV master variants, there are one or two ASVs that are much more prominent than the other ASVs on all the Islands, v+ or v-, while the majority of the 366 ASVs have a very reduced profile across the Islands. A final observation is that, by far, the lowest ASV diversity (number of unique variants, or Richness) is found on Graciosa, which is not surprising given the low number of DWV-positive samples from that island, and the greatest ASV Richness was found on São Miguel, while the ASV diversity of the remaining Islands is more defined by how these ASVs are distributed (Evenness, Shannon Index), both quantitatively and between the major DWV clades (Fig. 4, Supplementary Table S1).

The ASV distribution patterns were unique for each of the 102 colony samples (Fig. 3b; Supplementary Fig. S1; S2), providing both sufficient variability and sufficient replication to analyze how the colonies on the different Islands are related to each other using a Discriminant Analysis of Principal Components (DAPC) clustering approach (Fig. 3c and d). We explored two strategies for analyzing ASV data, based on different perspectives on how viral quasispecies evolve in response to adaptive challenges. The first strategy is based on the total evolutionary and adaptive ‘potential’ of the quasispecies, i.e. its full genetic breadth and depth, best represented here by the relative abundance of all 366 ASVs (Fig. 3c). The second strategy is based on the actual, ‘realized’ adaptation of the quasispecies in each colony, where out of all the ASVs available in each colony, a single ASV has become dominant through a combination of varroa, invasion history, chance, and selection. This strategy is best represented here by the relative abundance of the 20 single most abundant ASVs in the 102 colonies (Fig. 3d).

When considering the full quantitative diversity of all 366 ASVs, the DAPC produced three distinct island groups (Fig. 3c): Santa Maria and São Miguel, united primarily by the preponderance of ASVs from the DWV-C clade; Faial, with relatively high levels of ASVs from the DWV-B master clade; and a group including Pico, Flores, and Graciosa, which are dominated by ASVs from the DWV-A clade (Fig. 3a, middle rings; Fig. 3b).

When considering just the 20 dominant ASVs, the DAPC produced five distinct groups with different degrees of relatedness (Fig. 3d). One group included São Miguel and Santa Maria, which are practically superimposed through the “super-dominance” of ASV3, from the DWV-C clade, as the dominant ASV in most colonies on both these Islands (Supplementary Fig. S3). The proximity of Pico to Faial in the DAPC is mostly due to ASV-10, from the DWV-A clade, being the dominant ASV in many colonies on both Islands, while the slight separation of the Islands is due to the presence of different dominant ASVs from the DWV-B clade on Pico (ASV-23) and Faial (ASV-24) in a significant number of colonies on these Islands (Supplementary Fig. S3). ASV-1 and ASV-13 (DWV-A) are the “super-dominant” ASVs in most colonies on Flores, but only very rarely so on any of the other Islands, resulting in a clearly separate group of colonies from Flores, while ASV-13 was also the dominant ASV in both colonies on Graciosa, explaining the slight proximity of Graciosa to the Flores group (Supplementary Fig. S3).

### Deformed wing virus diversity and load are related to each other, but not to island varroa status

The adaptive capacity of a virus is highly dependent on the nature, extent, and distribution of genetic variation in its quasispecies. In this section, we analyzed several metrics of this diversity in relation to the varroa status of the Islands and the DWV loads in the colonies. These include “Richness” (a measure of the total absolute diversity available, *i.e.* the number of unique ASVs), “Evenness” (a measure of how evenly this diversity is distributed between the samples), and the Shannon-Wiener Index, which is one of several indices that combines aspects of Richness and Evenness into a single metric that can be analyzed. As intimated above, these diversity estimates varied greatly among colonies and Islands, with São Miguel (v-) and Faial (v+) exhibiting the highest median values in 2014/2015 (Fig. 4; Supplementary Table S1). However, there was too much variability between individual colonies and not enough difference between island groups in these metrics for any significant association between varroa status and DWV diversity, as shown by Bayesian inference modeling (Evenness: 53% probability; Shannon-Wiener index: 58% probability) and only a moderate probability for an increase in DWV diversity over time on v+ Islands relative to v- Islands (Richness: 84% probability; Table 1).

**Figure 5.**
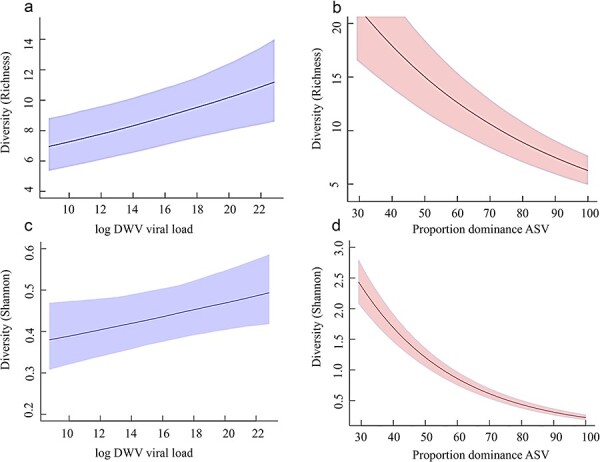
Bayesian generalized linear mixed model (GLMM) predictions relating ASV diversity (Richness, Shannon Index) to DWV log-loads (A; C) and to the proportion of the single most dominant ASV in each sample (B, D). The lines show the predicted means and the shaded areas show the 95% Bayesian CIs obtained from GLMMs (see Appendix 1 for modelling details).

DWV load is another factor that can affect both Richness (through the generation of *de novo* variation during replication; Domingo and Perales (2019)) and Evenness (through the preferential amplification of selectively advantageous, dominant, variants; Gisder et al. (2018); Ryabov et al. (2019)). Bayesian inference modeling identified evidence for both of these effects. First, there was a strong positive relationship between the DWV load and DWV genetic diversity, as measured by ASV Richness (total number of unique ASVs; Table 3; Fig. 5a), and the Shannon-Wiener index (Table 3; Fig. 5c), which contains a strong element of ASV Richness. However, these relationships were partly masked by the degree of dominance of the most abundant ASV in each sample. If ASV dominance was not accounted for, then the relationship between DWV load and DWV diversity was uncertain (Richness −0.013 ± 0.013; Shannon-Wiener 0.03 ± 0.022; Evenness 0.002 ± 0.005). However, when the degree of dominance (*i.e.* proportion) of the most abundant ASV in each sample was included in the model, then the relationship between DWV load and diversity was consistently positive for Richness and the Shannon-Wiener index, and moderately positive for Evenness (Table 3). This modeling revealed that the degree of dominance of the most abundant ASV in each sample had a strong negative relationship with all estimates of DWV genetic diversity (Table 3; Fig. 5). This relationship was expected for Evenness, dominance being diametrically opposite to Evenness (r = −0.95), but was also clearly important for ASV Richness (r = −0.63; Fig. 5b) and consequently also for the Shannon-Wiener index (r = −0.95; Fig. 5d).

**Table 3.** Model parameter estimates from Bayesian GLMMs relating measures of DWV diversity (Richness, Evenness, and the Shannon-Wiener index) explained by DWV log-loads and the degree of dominance of the single most dominant ASV in each sample (explanatory variables were mean-centred). Richness and Shannon-Wiener parameters are at the log-scale because a log-link was used in the modelling (Appendix 1). Estimates are presented with the means and standard deviations of the Bayesian posterior distributions as well as the Probability that their effect is in the direction indicated.

Response variable	Estimates	
**Richness**	Parameter	Probability
Intercept	2.180 ± 0.109	–
DWV load	0.033 ± 0.009	99.9% (positive)
Proportion dominant ASV	−0.018 ± 0.001	100% (negative)
**Evenness**	–	–
Intercept	0.238 ± 0.007	–
DWV load	0.0006 ± 0.001	65% (positive)
Proportion dominant ASV	−0.009 ± 0.0003	100% (negative)
**Shannon-Wiener index**	–	–
Intercept	−0.825 ± 0.076	–
DWV load	0.018 ± 0.009	98.5% (positive)
Proportion dominant ASV	−0.034 ± 0.002	100% (negative)

In summary, although both DWV load and DWV genetic diversity varied greatly across the Azores, and with each other, the varroa status of the Islands did not have a major influence on either of these sets of traits (Table 1).

## Discussion

These results provide several important novel insights into the relationships between honey bees, varroa, and the major and minor genetic variants of DWV. This includes how the introduction of varroa in geographically delimited areas impacts the genetic diversity and evolution of DWV communities, and how DWV genetic diversity can be used to reconstruct historical varroa invasion scenarios. In addition, these results extend the current knowledge on the invasion dynamics of honey bee pathogens in the Macaronesia region (Barroso-Arévalo et al. 2019, Lopes et al. 2022, 2023, 2024).

### A stable refuge for the rare Deformed wing virus-C master variant

The detection of DWV on varroa-free Islands in the Azores suggests that the virus was already present in the general Azorean honey bee populations prior to the arrival of varroa in 2000, most likely introduced through the trade in queens from mainland Europe during the 1980s, before the implementation of quarantine restrictions (Ferreira et al. 2020). However, one of the most interesting discoveries was the dominant presence (in terms of both prevalence and virus loads) of the extremely rare DWV-C master strain on two of the varroa-negative Islands: São Miguel and Santa Maria. We can only speculate as to how and when DWV-C first arrived on the Islands, but it is clear that the extreme isolation and varroa-free status of the Islands, facilitated by strongly enforced restrictions on honey bee movement to and between the Islands since the arrival of varroa, have been critical to the survival of DWV-C, even in the presence of both the DWV-A and DWV-B variants. The only other record of DWV-C dominance in honey bee colonies is from Devon, a relatively insular county in England, particularly during spring (when varroa-levels are naturally low), before being superseded by DWV-A and DWV-B later in the season (Mordecai et al. 2016, Kevill et al. 2017, 2019).

### Deformed wing virus-A, -B, and -C may have distinct relationships with varroa that could help explain their relative dominance on different Islands

Although DWV has only become a global phenomenon through its association with varroa, the results presented here for DWV-C are the most convincing evidence to date that as a virus species, DWV is eminently capable of surviving and even thriving in honey bees without varroa-mediated transmission. This is a key novel insight into the ecology and adaptive evolution of DWV and its relationships with honey bees and varroa. While DWV-C was also technically present on the varroa-infested Islands, quantitatively it only persisted there at extremely low levels on the outer margins of quasispecies dominated by DWV-A and DWV-B master variants (Supplementary Fig. S2). There are several possible explanations for these contrasting observations. Both DWV-A and DWV-B are highly dependent on varroa-mediated transmission in order to persist at relatively stable loads in the honeybee population (Martin et al. 2012, Mondet et al. 2014, Ryabov et al. 2014, Norton et al. 2021, Woodford and Evans 2021, Doublet et al. 2024) and their titres will rapidly drop to background level as soon as varroa is removed from the colonies (Locke et al. 2017). This is the theoretical foundation for practical varroa management worldwide (Traynor et al. 2020) and supported in the current study by the very low DWV loads in those few colonies on the varroa-free Islands São Miguel, Santa Maria, and Graciosa that were dominated by DWV-A and DWV-B (Supplementary Table S1). DWV-C, on the other hand, can apparently efficiently generate high colony-level titres without the help of varroa, through alternative transmission routes. While this explains the dominance of DWV-C on the varroa-free Islands, it does not easily explain its relative absence from the varroa-infested Islands. There are two broad hypotheses that can be considered. The first is that DWV-C is vastly inferior to DWV-A and DWV-B in its adaptation to varroa-mediated transmission, and is rapidly displaced from the quasispecies through positive selection for DWV-A and DWV-B, driven by their preferential and highly efficient varroa-mediated transmission. Such ecological succession and genetic adaptation of different viruses when subjected to a novel vector has been extensively documented in many host–vector–virus systems (Strauss et al. 2012, Chinchio et al. 2020, Islam et al. 2020), including honey bees and varroa (Sumpter and Martin 2004, Martin et al. 2012, Mondet et al. 2014, Hasegawa et al. 2023), exemplified most recently by the apparent worldwide displacement of DWV-A by DWV-B (Norton et al. 2020, Paxton et al. 2022). The other main hypothesis assumes that DWV-C is similarly transmitted by varroa as DWV-A and DWV-B, but is too virulent under these circumstances at individual and/or colony level, and gradually disappears from the quasispecies through attrition, bee mortality, and negative selection, similar to other excessively virulent varroa-transmitted viruses and virus strains (Sumpter and Martin 2004, Mondet et al. 2016). At an individual level, this involves a range of adult honey bee hygienic behaviors that can detect and remove diseased brood, including varroa-infested pupae (Mondet et al. 2016, Oddie et al. 2018, 2021, Gabel et al. 2023). One of the triggers for this is varroa-induced virus damage (Mondet et al. 2016), with lower or slower individual (varroa-transmitted) virus virulence having a better chance of escaping this hygienic behavior at individual level, thus becoming dominant at colony level (Kevill et al. 2017). A similar process also operates at colony level, with winter mortality selecting against excessive colony-level virulence, allowing less virulent strains to become dominant at apiary or population level (Sumpter and Martin 2004, Ryabov et al. 2014, Norton et al. 2021, Paxton et al. 2022). Naturally, these hypotheses are not necessarily mutually exclusive, and reality may well include elements of both. The contrast between the presence of some DWV-A and DWV-B dominant colonies on the varroa-free Islands, compared to the near-complete absence of DWV-C from varroa-infested Islands, both at colony level and within the quasispecies (Supplementary Fig. S2), suggests that the selection for DWV-A and DWV-B (or against DWV-C) in a varroa context is much stronger than the selection for DWV-C (or against DWV-A/B) in a varroa-free context. This in turn may simply reflect the elevated potency of varroa-mediated transmission for inducing damage, and thus as a selective force (positive or negative), relative to other transmission routes (Möckel et al. 2011, Norton et al. 2021).

The relationship between DWV-A and DWV-B in this study is more balanced, although DWV-A appears to still dominate at most levels, contrary to worldwide trends (Paxton et al. 2022), both quantitatively and in terms of diversity. The same hypotheses and processes outlined above with respect to DWV-C would also have shaped the distributions of DWV-A and DWV-B, just with less drastic outcomes. Both strains have a long historical association with varroa-mediated transmission in honeybees (Wilfert et al. 2016, Hasegawa et al. 2023, Doublet et al. 2024). DWV-A is generally the main initial variant, with DWV-B emerging and displacing DWV-A at a later stage. There is uncertainty as to whether DWV-A or DWV-B is more virulent at individual bee level (Ryabov et al. 2014, 2019, McMahon et al. 2016, Gisder et al. 2018, Tehel et al. 2019); Norton et al. 2020) or colony level (Moore et al. 2011, Ryabov et al. 2014, Natsopoulou et al. 2017). Although both DWV-A and DWV-B can replicate in mites (Damayo et al. 2023), DWV-B appears to be more efficient at this (Gisder et al. 2021) and is generally better adapted to varroa-mediated transmission than DWV-A (Gisder et al. 2021, Norton et al. 2021), allowing it to persist longer and accumulate titers faster in individual honey bees (Gisder et al. 2018, Ryabov et al. 2019, Norton et al. 2020) and bee colonies (Norton et al. 2021). These relative differences in basic properties between the major DWV strains can also be selected for, or against, by beekeeping practices (Brosi et al. 2017, Ray et al. 2023), with the increasing frequency and intensity of varroa treatment to enhance colony winter survival inadvertently also allowing increasingly virulent varroa-associated virus assemblages, that should have perished with the colony, to survive winter in the few mites that escape or are resistant to acaricide treatment (Mitton et al. 2022), providing furthermore also an efficient genetic bottleneck to accelerate adaptation. DWV-B’s superior persistence and replication in varroa mites during long broodless periods would be a clear advantage in this scenario. This means that different master strains may therefore dominate at different times during the season (Kevill et al. 2017), depending on the status of the honey bee colony, the transmission properties of the major strains, the efficiency of honey bee hygienic behavior, and the type, timing, frequency, and effectiveness of varroa control (Gabel et al. 2023), and highlights the dynamic and continuously evolving status of the relationships between honey bees, varroa, and their viruses (Neumann et al. 2012, Paxton et al. 2022, Doublet et al. 2024).

### New insights into the biogeographic origin of Deformed wing virus

The stable persistence of DWV-C in the absence of varroa-mediated transmission also has important implications for the possible origins of DWV. One hypothesis (the “endemic” hypothesis) is that DWV is a globally distributed endemic virus, persisting in local wild and managed bees, other hymenopterans, and perhaps even other insect families at sublethal background levels ahead of the varroa expansion front, and only emerging as a honey bee disease behind the varroa expansion front (Neumann et al. 2012). The main driver for this hypothesis is that DWV has regularly been detected in honey bees beyond the varroa expansion front, such as in isolated/island regions in Scandinavia (Forsgren et al. 2012, Doublet et al. 2024), Newfoundland (Shutler et al. 2014), Colonsay island (Fürst et al. 2014, Ryabov et al. 2014), the Isle of Man (Fürst et al. 2014, McMahon et al. 2015), the Channel Islands (Manley et al. 2019), and the Hawaiian Islands of Kauai and Maui (Martin et al. 2012, Grindrod et al. 2021), and that it replicates and persists in honey bees and other bee species in a largely well-adapted, stable manner (Grozinger and Flenniken 2019, Tehel et al. 2020, Yañez et al. 2020b, Woodford and Evans 2021). However, this has almost always been within the context of permitted transport across the varroa front of queens, eggs, and semen, all of which can harbor significant levels of DWV (Yañez et al. 2020b). The main alternative hypothesis (the “co-dispersal” hypothesis) is that DWV originated in southeast Asia and spread through *A. mellifera* populations worldwide together with varroa (Hasegawa et al. 2023), after the initial adaptation and subsequent speciation (Anderson and Trueman 2000) of varroa from *A. cerana* to *A. mellifera* in mixed apiaries in southeast Asia during the Green Revolution of the 1950s–1960s (Traynor et al. 2020). This distinction is important, particularly with respect to the possible consequences of spill-over of DWV into local wild bees and other insects that either may (“endemic”) or may not (“dispersal”) have had prior exposure, tolerance, or resistance to the virus (Strauss et al. 2012, Chinchio et al. 2020). The ability of DWV-C to persist and even thrive in *A. mellifera* in the absence of varroa-mediated transmission is problematic for both these hypotheses, but particularly for the endemic hypothesis. This is because of the well-established absence of DWV in long-standing isolated honey bee populations, such as the Chatham Islands (Mondet et al. 2014); the Åland Islands (Thaduri et al. 2019, Doublet et al. 2024); and, perhaps most conclusively, in the extensive pre-varroa surveys in Australia (Wilfert et al. 2016, Roberts et al. 2017) and New Zealand (Mondet et al. 2014), both large island nations with extensive, self-sustaining, and import-free beekeeping sectors; extremely strict, culturally deeply engrained quarantine systems concerning all manners of biological threats; and, until recently (Mondet et al. 2014, Lester et al. 2022, Chapman et al. 2023), completely varroa-free, despite extensive surveillance of its borders, harbors, and airports (Todd et al. 2007, Chapman et al. 2023). If DWV is an old endemic virus of honey bees that can sustain itself independent of varroa, then it should have been present, as DWV-C, in these populations.

However, DWV-C is also problematic for the dispersal hypothesis since it predicts the presence of stable, self-sustaining DWV in varroa-free honey bee populations, either those ahead of the varroa expansion front where cross-front transfer of queens, eggs, and sperm is permitted or well-managed beekeeping operations behind the front with low levels of varroa and minimal competition from the varroa-transmitted DWV-A and DWV-B strains. In the present study, this scenario was found on Santa Maria and São Miguel, but not on Terceira, São Jorge, or Graciosa, despite similar histories, varroa-free conditions, and the transfer of honey bees and queens between the Islands during the 1980s (see later) for DWV-C to become established. DWV-C was also detected in historical varroa samples (Hasegawa et al. 2023), as well as in isolated pockets in England (Mordecai et al. 2016, Kevill et al. 2017, 2019), Brazil (De Souza et al. 2019), and the USA (Kevill et al. 2019), showing that it can survive within a varroa environment, similar to its presence on the varroa Islands of the Azores (Flores, Pico, and Faial). For both origin hypotheses, therefore, it is the absence of DWV-C in places where it might be expected that is problematic. The dispersal hypothesis is thus favored because it can frame this absence within the context of varroa transmission, the extreme competition from DWV-A and DWV-B, and the possibility of the gradual extinction of DWV-C, as is thought to have happened to its closest relative, DWV-D (De Miranda et al. 2022). Why DWV-C persists abundantly on certain varroa-free Azorean Islands but not on others remains a mystery, but it matches the general incidence and relative abundance patterns of DWV-C worldwide.

### Deformed wing virus diversity is shaped more by virus load than by varroa presence

Viruses generate new variation through error-prone replication, creating a broad link between genetic diversity and virus load. Recombination between strains, most likely through template switching during replication (Dolan et al. 2018), can further re-assort this micro-variation into still higher levels of genetic diversity within the quasispecies, with recombinants often at a selective advantage over both parent strains (Moore et al. 2011, Ryabov et al. 2014). Both *de novo* error generation and recombination are therefore super important for the vitality of a quasispecies (Dolan et al. 2018). Selection then acts to reduce this variation through preferential amplification or transmission of preferred variants, disrupting this broad link. Variation can also disappear through bottleneck events, either from low loads or during transmission. The effects of all these processes were clearly demonstrated by the quantitative dominance of a few selected amplicon sequence variants (ASVs) within a broad background diversity of ASVs across all three DWV clades, with the greatest genetic richness in clade DWV-B, followed by DWV-A, and DWV-C, which conforms to similar patterns described elsewhere (Hasegawa et al. 2023). Previous reports, using melting curve analyses, have linked high DWV diversity to low virus loads (Martin et al. 2012). The strong effect of ASV dominance on diversity shown here suggests that these earlier links may reflect more the extremely asymmetrical distribution of this variation at higher loads, through the replication-selection processes described above, and that the underlying genetic richness is positively related to loads. Finally, the discovery of high DWV loads in the absence of varroa transmission allowed us to uncouple the effects of load (replication) from varroa (transmission) on DWV diversity, with the latter contributing minimally. DWV genetic diversity is largely driven by error-prone replication and asymmetrical selection during load generation, independent of the mode of transmission.

### Reconstruction of Deformed wing virus and varroa invasion history using Deformed wing virus genetic information

**Figure 6.**
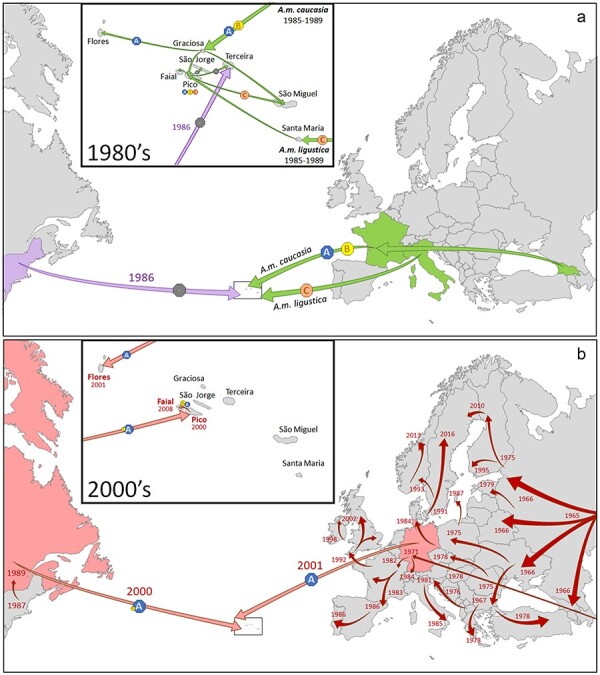
Reconstruction of the historical introduction of the DWV major strains DWV-A (blue hexagon), DWV-B (yellow hexagon) and DWV-C (orange hexagon) to the different Azores islands through: (a) The import, hybridization on Pico and dispersal to the remaining Azores islands of mated queens (green arrows) and package bees (purple arrows) during the pre-varroa late 1980’s, with the grey hexagons indicating bee movements without introducing DWV, and (b) The accidental introduction of varroa to Flores, Pico and Faial (pink arrows) during the early 2000’s, set against the historical record of the varroa dispersal through Europe and North America (red arrows). References for the European and North American varroa dispersal are: De Jong *et al.* (1982) for all records up to 1978 (former USSR, Eastern Europe, Greece, Turkey, Germany); Hansen (1984) for Denmark; Silva de Oliveira *et al.* (2021) for Sweden through 2016; Oddie and Dahle (2021) for Norway; Kauko (1992) for Finland; Doublet *et al.* (2024) for Northern Scandinavia through 2013; Asten *et al.* (2019) for Estonia; Paxton (1992) for England; Coffey *et al.* (2013) for Ireland; Wenner and Bushing (1996) for the USA; Sobkowich *et al.* (2022) and Doublet *et al.* (2024) for Canada; Diller and Imdorf (2009) for Switzerland; Colin *et al.* (1997) for France; Frilli (1988) for Italy; Ministerio de agricultura, pesca y alimentación (1986) for Spain; Belchior (1996) for Portugal. The map of the Azores was downloaded for free academic use from www.pptmaps.com.

The close, almost exclusive, relationship between DWV and varroa opens up the possibility to use DWV genetic diversity to track varroa invasion history, independent of (or complementary to) the official documentation. The use of virus genetic diversity and phylogenetics to track epidemics (Grubaugh et al. 2019) or host migration (Kitchen et al. 2008) has been well established, most recently during the SARS-CoV-2 (COVID-19) epidemic (Turakhia et al. 2020, Nelson 2021). Obviously, such tools work best with contemporaneous samples when the lineages are fresh and unpolluted by subsequent evolution. However, virus quasispecies are also a cumulative repository of historic variants and diversity, propagated along with their currently dominant descendants (Dolan et al. 2018). This deep-lying genetic diversity could theoretically be used to reconstruct the DWV and varroa invasion history in the Azores, 40+ years after the event. There are two separate invasions to consider: a primary invasion by DWV, accounting for the presence of DWV on the varroa-free Islands (Fig. 6a), followed by one or more varroa invasions (Fig. 6b). Between 1985 and 1989, a breeding program was implemented to improve the local honeybee genetic stock by importing *A. m. ligustica* queens from Italy to Santa Maria and *A. m. caucasia* queens to Graciosa, which were produced in France but from breeder queens imported from Georgia in the Caucasus (Anonymous 1987, Ferreira et al. 2020). During this time, varroa was already well established throughout the USSR, Eastern Europe, and Caucasia, through internal transport of varroa-infested honey bees and queens from the Primorsky region in Eastern Siberia (Alpatov 1976, Crane 1978) (Fig. 6b), and was rapidly spreading through Western Europe, including Italy (first detection in 1981 in Friuli-Venezia Guilla, from Yugoslavia; (Frilli 1988), and France (first detection in 1982 in Alsace, from Germany; (Colin et al. 1997)). The *A. m. ligustica* and *A. m. caucasia* subspecies were subsequently crossbred on Pico, with the hybrid queens distributed to the other Islands, with particularly high acceptance on São Miguel (Fig. 6a). These honey bee breeding initiatives probably brought DWV (but not varroa) to the Azores, with the *A. m. caucasia* (from varroa-infested Georgia, through France to Graciosa) possibly dominated by DWV-A and DWV-B variants, and the *A. m. ligustica* from Italy possibly dominated by DWV-C variants, which subsequently found their way to São Miguel via the hybrid queens from Pico (Fig. 6a). This would explain both the proximity of São Miguel to Santa Maria and the proximity of Graciosa to Pico in the total ASV DAPC (Fig. 3c).

The subsequent arrival of varroa on Pico in 2000 (Fig. 6b) re-configured the local DWV population through selection for both existing and newly imported DWV-A and DWV-B variants, causing Graciosa to drift away from Pico in the dominant ASV DAPC (Fig. 3d), with varroa transmission being the main driver behind the dominant ASVs on the varroa-infested Islands. The historical records in the Azores suggest that varroa arrived independently on Pico (in 2000, most likely from Canada) and Flores (in 2001, most likely from Germany), with a subsequent secondary migration to Faial (in 2008, most likely from Pico), which are geographically close enough for this to occur naturally, through swarms, possibly facilitated by the frequent ferries between these Islands. This scenario is best represented by the dominant ASV DAPC (Fig. 3d), which places Pico and Faial in close proximity and away from Flores, largely due to the nature of the dominant DWV-A ASVs on these three Islands. These results suggest that DAPCs based on dominant DWV ASVs, selected through varroa transmission, may be more suitable for modeling varroa invasions, while DAPCs based on the full DWV genetic diversity may be more suitable for reconstructing strictly virus invasions.

## Conclusion

In conclusion, this research not only adds to our understanding of the global distribution of DWV but also strengthens the role of varroa in altering the DWV genetic diversity landscape and our understanding of the unique relationships of the main DWV variants with varroa. This research also confirms the scientific value of island archipelagos, such as the Azores, as possible refuges of unique material, time capsules of historical events, and natural laboratories for investigating evolutionary processes and outcomes in real time. Therefore, the heterogeneous distribution of pathogens in the Azores archipelago constitutes a clear example of how geographic isolation acts as a shield and how the global trade of honey bees facilitated by human movements can pose a serious threat to these populations and trigger their evolution.

## Materials and methods

### The history of honey bees and varroa in the Azores archipelago

The Azores is the northernmost archipelago of Macaronesia and has been managed under Portuguese sovereignty since the fifteenth century. During human colonization, the settlers introduced many plant and animal species, including the honey bee. Genetic analyses have shown that these historical honey bee introductions came from the north-western side of Iberia, and that the Azorean honey bees therefore descended from *A. m. iberiensis* (Ferreira et al. 2020). Another important honey bee migration wave occurred in the 1980s, when the regional agricultural authority launched a breeding program fully relying on subspecies imported from queen breeders in France and Italy (Ferreira et al. 2020; Fig. 6a). Since then, and until the end of the twentieth century, some beekeepers continued to occasionally import exotic queens from varying geographical origins. Due to this lack of oversight, varroa managed to invade three Azorean Islands at the beginning of the twenty-first century through the illegal importation of honey bee queens (Fig. 6b). In fact, human-assisted honey bee transport, whether deliberate or accidental, has been the cause of nearly every breach of varroa quarantine throughout its short history, including continental Europe (De Jong et al. 1982), Scandinavia (Silva de Oliveira et al. 2021; Doublet et al. 2024), the British Islands (Paxton 1992, Coffey et al. 2013), South America (De Jong et al. 1982), North America (Wenner and Bushing 1996, Sobkowich et al. 2022, Doublet et al. 2024), Hawaii (Martin et al. 2012), New Zealand (Mondet et al. 2014), and recently the Åland Islands (Finnish Food Authority 2021), L’Ile d’Ouessant (Ménage and L’Hostis 2021), and Australia (Chapman et al. 2023). In the Azores, varroa was first sighted on Pico in 2000, on Flores in 2001, and on Faial in 2008 (Ferreira et al. 2020). The remaining six Islands have recently been declared mite-free by the European Union (European Commission, E 2019).

### Experimental design

This study was designed around several unique and key features of beekeeping in the Azores. These included long-term, sustainable, and largely isolated honey bee populations on eight of the nine Islands (Corvo had no honey bees until 2015); large bodies of water separating the Islands from the mainland and from each other; a strong sense of identity, self-sufficiency, and cultural independence of the Azorean people facilitating a highly effective restriction on honey bee movement to and between the Islands since early 2000; and an excellent historical record of varroa invasion (2000 in Pico, 2001 in Flores, and 2008 in Faial), resulting in three comparable groups of Islands with both varroa and DWV present, just DWV present, or neither varroa nor DWV present. This experimental structure, combined with a large volume of data collected across two well-separated time intervals (2014/2015 and 2020), permitted a reliable analysis of the quantitative and genetic changes in the DWV population over time, in relation to the presence or absence of varroa and in relation to the known historical transfers of honey bees and varroa to and between the Islands.

### Sample collection

A total of 494 adult honey bee samples were collected from individual colonies in 176 apiaries distributed across eight Islands, including three varroa-infested (v+) Islands (Pico, Flores, and Faial) and five varroa-free (v-) Islands (Santa Maria, São Miguel, Terceira, São Jorge, and Graciosa). Most of the apiaries were represented by three colonies (126 apiaries) or two colonies (39 apiaries), with the remaining apiaries contributing anywhere between one (13) and six (1) colonies (Supplementary Table S3). Only six apiaries were sampled both in 2014/2015 and 2020: three on São Jorge and one each on Faial, Santa Maria, and Terceira, although different colonies were sampled from these apiaries on different occasions. The main sample collection occurred during July and August of 2014 (189 samples from São Miguel, Terceira, Graciosa, and São Jorge) and 2015 (213 samples from Santa Maria, Pico, Faial, Flores, and again São Jorge), followed by a more limited re-sampling of a few Islands during 2020 (92 samples from Santa Maria, Terceira, Faial, and São Jorge) (Supplementary Fig. S4). For each colony, approximately 150 worker honey bees (non-reproductive females) were collected from the outside frame of the brood and placed alive in ventilated cardboard boxes supplemented with candy for subsequent transportation to the analytical laboratory in Bragança, on the Portuguese mainland. The 494 samples were stored at −80ºC until molecular analysis.

### Deformed wing virus genetic diversity and meta-barcoding RT-qPCR assay design

In order to analyze the DWV genetic diversity in the Azores, a novel RT-qPCR assay was designed that would amplify a relatively variable region of the DWV genome (in order to have enough diversity to analyze) of about 200–250 nucleotides (the optimum size range for MiSeq2 meta-barcoded amplicon sequencing) that was flanked by highly conserved regions (to ensure that as much of both known and potentially novel major and minor DWV genetic diversity would be amplified), using primer design strategies and principles outlined previously (De Miranda et al. 2013). The assay was designed and used well before the DWV-D sequence was elucidated and published (De Miranda et al. 2022), and was based on an alignment of the reference genomes for DWV-A (AY292384), DWV-B (AY251269), and DWV-C (ERS657949), together with numerous minor variants. A 210 nucleotide region between nt 8621 and 8830 of the DWV-A reference genome (AY292384) in the DWV RNA-dependent RNA polymerase (RdRp) gene (itself the most conserved and stable gene of the DWV genome) was identified satisfying these primary criteria. Subsequently, the target-specific components of the meta-barcoded DWV amplification primers were designed, such that the two 3ʹ terminal nucleotides of both the forward and reverse primers were located in the first and second codon positions of relatively conserved amino acids (Leu and Asp, respectively) in the RdRp region in question, avoiding the degenerate third codon position (and thus potential primer-template mismatches). The reverse primer works equally well for DWV-D (MT504363), which is identical in sequence to DWV-B and DWV-C in that region. However, the forward primer now has a mismatch with the DWV-D consensus sequence at the −3 position from the 3ʹ terminus, within the critical range where mismatches can affect polymerase binding and primer extension (Wu et al. 2009). There are also mismatches between the forward primer and DWV-C and between the reverse primer and both DWV-C and DWV-B, but these are far away from the 3ʹ terminus, with negligible risk of non-amplification (Wu et al. 2009) and minimal effect on potential technical biases in ASV distribution between the major strains (Green et al. 2015). The thermodynamic parameters of the qPCR protocol were also deliberately designed to be permissive of template-primer mismatches, with generous annealing and extension times and a moderate annealing temperature. The risk of non-specific amplification products with such a permissive thermodynamic profile was minimized by reducing the primer concentrations to 0.2 µM and avoiding the most obvious primer-dimer pitfalls, such as CG dinucleotides at the 3ʹ end of either primer.

The effect of the PCR assay design on the distribution of major and minor DWV variants in the samples is unknown, and in some respects not very relevant. There is natural variation across the DWV genome, also in the conserved regions, so that primer-template mismatches (and potential distributional biases) are inevitable for a subset of genomes in all samples, no matter what major strain. The only true concern would be for DWV-D variants, where the mismatch in the forward primer is sufficiently close to the 3ʹ end to threaten non-amplification, and thus non-detection of DWV-D, should it exist in these samples. However, since the mismatch is located on a degenerate third codon position, with all alternative nucleotides neutral with respect to the coding of the amino acid in question (proline), the likelihood is that if DWV-D was indeed present in these samples, the natural variability in this mismatched third codon position would have allowed at least a subset of correctly matched DWV-D genomes to be amplified, thus avoiding complete non-detection, though perhaps returning a biased distribution. Results elsewhere, using target-free amplification and detection approaches, have shown that DWV-D is currently most likely extinct throughout the world, and has only been detected in a singular sample from 1970s Egypt, before the arrival of varroa (De Miranda et al. 2022). Its absence from these samples is therefore not at all unusual, and probably genuine.

### RNA isolation, cDNA synthesis, and qPCR

For each of the 494 samples, total RNA was extracted from a pool of 30 workers using the Monarch Total RNA Miniprep kit (New England Biolabs Inc., MA, USA). All steps were performed on ice to preserve the RNA integrity. Prior to extraction, the 30 workers were transferred into a sterile double bag strainer (BA6040, Seward, Worthing, UK) with 6 mL of cool DEPC water (E476, VWR, PA, USA) and then homogenized using the MixWell Lab Blender (Alliance Bio Expertise, Guipry-Messac, FR) for two cycles, each of 60 s, with a 30-s break in between. A volume of 500 µL of the tissue homogenate was combined with 800 µL of the Monarch RNA Lysis Buffer into a 2-mL tube containing two zirconia beads of 3 mm and placed in a Precellys apparatus (Bertin Instruments, Montigny-le-Bretonneux, FR) for mechanical tissue disruption with the following protocol: 6200 rpm; 5 s; three times. Each sample was then centrifuged for 2 min at 16 000 ×g at 4ºC to pellet the debris, and 800 µL of the supernatant was transferred to a gDNA removal column fitted with a collection tube. The subsequent steps of RNA extraction followed the instructions detailed in the Monarch Total RNA Miniprep protocol—Part 2, without modifications. The RNA extracts were normalized to a concentration of 250 ng/µL with nuclease-free water and stored at −80°C until reverse transcription. The cDNA of each sample was synthesized 24 h after RNA extraction using an iScript^TM^ cDNA Synthesis Kit (Bio-Rad, CA, USA) with 1 µg of RNA in 20 µL reactions, following the manufacturer’s instructions. The Bio-Rad iScript^TM^ cDNA Synthesis Kit uses a blend of random hexamer and Oligo-dT primers for priming the reverse transcription reaction. The cDNAs were stored at −20°C until analysis. The 494 cDNA samples were screened for DWV by qPCR performed on a QuantStudio 5 apparatus (Applied Biosystems, MA, USA) using SYBR Green chemistry. Each qPCR reaction was carried out in a 10-µL total volume, containing 3 µL of the 1:10 diluted cDNA, 5 µL of 2X iTaq Universal SYBR Green Supermix (Bio-Rad, CA, USA), and 500 nM of each primer (Supplementary Table S4). The amplification profile consisted of an initial denaturation step at 95°C for 30 s followed by 40 cycles of denaturation at 95°C for 15 s, annealing at 56°C for 20 s; extension at 60°C for 30 s, and fluorescence reading. Amplification was immediately followed by a Melting Curve Analysis consisting of 60 s at 65ºC; followed by increasing the temperature by 0.5ºC intervals for 5 s followed by fluorescence reading, ending at 95ºC, in order to confirm the veracity of the amplified product. The DWV Cq values of the samples were converted to copies DWV/reaction using a 7-step 10-fold dilution series of external reference standards, and subsequently to copies/bee using the various dilution factors incurred during the RNA extraction and cDNA synthesis.

### Illumina amplicon sequencing

A DWV amplicon sequencing library was constructed from all 110 DWV-positive RNA samples from the three varroa-infested Islands (v+: Pico, Faial, and Flores) and the three DWV-positive varroa-free Islands (v-: Graciosa, São Miguel, and Santa Maria) using a two-stage amplification process. The first stage (PCR-1) involved a short, high-fidelity reaction using DWV primers that were modified to include the Illumina sequencing adaptors as well as one to four random nucleotides (nt) to improve the quality of the sequence reads (Wu et al. 2015). The PCR-1 reactions consisted of 5 µL of Q5 High-Fidelity 2X Master Mix (New England Biolabs Inc., MA, USA), 0.5 µL of each primer pool (Supplementary Table S4) at a concentration of 6 µM, 1 µL of DEPC water, and 3 µL of the cDNA extract previously diluted at 1:10. The PCR-1 reactions were performed in the T100 thermocycler equipment (Bio-Rad, CA, USA) with the following protocol: 98ºC for 30 s, 35 cycles of 98ºC for 10 s, 54°C for 30 s, and 72ºC for 20 s, and a final extension of 72ºC for 2 min. In this step, eight samples in which qPCR amplification was close to the limit of detection failed the amplification on qualitative PCR. Therefore, only 102 samples proceeded to the next step. After confirming the successful production of the desired amplicon by PCR-1, the amplicons were subjected to a second indexing PCR reaction (PCR-2) to incorporate the unique 7-nt sequence indexes and the Illumina-specific adaptors P5 and P7 required for library sequencing. These reactions were carried out in a 14-µL total volume containing 7 µL of the KAPA HiFi HotStart ReadyMixPCR Kit (Kapa Biosystems, MA, USA), 0.7 µL of each unique index at 1 µM, 2.8 µL of water, and 2.8 µL of the amplicon. The indexing thermal cycling profile consisted of 95ºC for 3 min, 10 cycles of 95ºC for 30 s, 55ºC for 30 s, and 72ºC for 30 s, and a final extension of 72ºC for 5 min. After indexation, the PCR products were purified using 0.8x AMPure XP beads (Agencout, Beckman Coulter, MA, USA). Small samples of the indexed amplicons were first run in a 1% agarose gel for quality control and used to quantify the DNA concentration in the Epoch Microplate Spectrophotometer (Agilent-BioTek Instruments, CA, USA), after which each sample was normalized to a final concentration of 20 nM, and pooled with all the other samples. As per standard practice for metabarcoding studies, the negative controls from each of the molecular steps were included in all subsequent steps through to the final pooled sequencing library, as a set of quality controls in the final data set for possible contamination during the molecular procedures (Bell et al. 2019, 2023, Wilding et al. 2023). The pooled library was assessed for amplicon size distribution on a TapeStation 2200 using the D5000 Kit (Agilent Technologies, Inc., CA, USA) and quantified by a SYBR green qPCR assay using a KAPA Library Quantification Kit (Kapa Biosystems, MA, USA). The library was denatured and diluted as recommended by Illumina and loaded at 12 p.m. on a MiSeq flow cell spiked with 10% PhiX. The final library was sequenced on the Illumina MiSeq platform (Illumina, Inc., CA, USA) using the 2 × 250 cycles v2 chemistry, according to the manufacturer’s instructions.

### Bioinformatic analyses

The sequencing reads were de-multiplexed in the Illumina BaseSpace Sequence Hub according to their unique indices incorporated during library construction, which generated two FASTQ files per sample: R1 and R2. These sequences were subsequently uploaded to the Galaxy platform (usegalaxy.org; Afgan et al. 2018) and processed with Mothur (Schloss et al. 2009) following the standard operating procedure for MiSeq data (Batut et al. 2018, Hiltemann et al. 2023a, 2023b), with minor modifications. Briefly, the contigs of the paired-end R1 and R2 were constructed using the *make.contigs* command, and the primer sequences, along with the 1–4 random nucleotides, were removed using the *Cutadapt* command. Next, the paired-end reads were subjected to the first data cleaning step by removing those of poor quality using the *screen.seqs* command (minlength = 169, maxlength = 212, maxambig = 0). The reads that passed this filter were then analyzed using the *unique.seqs* and *count.seqs* commands, which generated a *FASTA* file containing the unique sequences for each sample as well as a summary table displaying the number of reads detected for each unique sequence in each sample. The unique sequences [also known as amplicon sequence variants (ASVs)] were denoised by using the *shhh.seqs* command (Quince et al. 2011), and chimeric sequences were filtered out by the *chimera.vsearch* command. All unique sequences were queried through BLASTn [nucleotide collection (nr/nt) performed in July 2022] to remove non-specific sequences. A total of 1 571 140 reads were generated from the 102 samples included in the Illumina sequencing run. The paired-end reads were filtered as follows: 21.6% were of poor quality, 0.9% were chimeric, and 0.6% were not DWV-specific. Furthermore, only unique ASVs with more than five reads were retained in order to avoid including cross-sample contaminants or ASVs containing technical mutations, introduced by the molecular procedures (Bell et al. 2019, 2023, Wilding et al. 2023), while still allowing the depth of the natural DWV quasispecies diversity, as represented in part by such low-level ASVs, to be explored. A total of 1 205 315 sequence reads (76.7%) passed all these stringent filters. These corresponded to a total of 366 unique ASVs identified from the 102 DWV samples, with a median of 10 426 (± 697) reads per sample. Of these 1.2 million reads, only 12 reads were from the technical negative controls: 8 reads corresponding to 7 of the most abundant ASVs found in the biological samples (ASV1, ASV3, ASV13, ASV23, ASV24, ASV29, ASV33), representing the incidence of cross-contamination between the samples, and 4 reads corresponding to 4 ASVs only found in the technical controls, representing the incidence of ASVs containing technical mutations. This corresponds to a cross-contamination rate of about 1 read per sample for the most abundant ASVs, down to about 0.001 read per sample for the rarer ASVs. Increasing the threshold to >10 reads per ASV would only remove another 38 ASVs from the dataset, whose reads are furthermore mostly confined to single individual samples. Their removal would not significantly improve the accuracy of the statistical analyses, which are mostly driven by high abundance ASVs that are present in many samples (Wilding et al. 2023).

### Phylogenetic, clustering, and discriminant analyses

The phylogenetic trees were constructed in Mega X (Kumar et al. 2018) using all 366 ASVs (Fig. 2) as well as the most abundant ASVs identified in the 102 DWV-positive colony samples (Supplementary Fig. S3). The ASVs were aligned with GenBank reference sequences for DWV-A (AY292384), DWV-B (AY251269), DWV-C (ERS657949), and DWV-D (MT504363) using the Clustal*W* model (Thompson et al. 1994). The consensus maximum likelihood trees were constructed from 1000 bootstrap replicates, and Tamura 3-parameter (Tamura 1992) was the best evolutionary model identified by MEGA X. Darwin bee virus-3 (MG995697) was used as an outgroup.

After classifying the 366 ASVs using the alignments with the reference sequences, the relative abundances of the DWV master variants were calculated from all sequence reads identified in each colony sample.

The genetic structure of the DWV populations in the Azores was inferred from either the single most abundant ASV found in each of the 102 sequenced samples or from the full quantitative diversity of all 366 ASVs in each sample using DAPC (Jombart et al. 2010) in the *adegenet* (Jombart 2008) package for R version 4.2.2 (R Core Team 2022).

### Statistical and Bayesian inference analyses

No mortality data were available for the sampled colonies through to the following spring as a possible trait that could be affected by DWV amount and/or strain composition (Yañez et al. 2020b). The varroa phoretic infestation rate, a more immediate factor that could in particular affect the DWV strain distribution (Paxton et al. 2022, Doublet et al. 2024), could also not be used in the analyses, since many the samples from the v+ Islands were taken during varroa treatment, thus nullifying the explanatory value of any infestation rate data obtained from the samples. Although in many respects DWV load is a reasonable proxy for varroa burden, this obviously only applies for the varroa-infested colonies and Islands. This left only the island varroa status as a usable “varroa” variable for explaining certain trends in the virus data. To estimate the effect of the varroa infestation status of Islands on DWV prevalence, loads, and genetic diversity at the colony level, we used a Generalized Linear Mixed Model (GLMM) framework in which varroa infestation status and sampling year were fixed effects and apiary identity was included as a group-level random effect on the intercept. We used the number of reads for each of the 366 DWV-ASVs in each sample as our dataset for calculating the various DWV genetic diversity estimates for each sample. DWV genetic diversity was represented by “Richness” (the number of unique ASVs in a sample), “Evenness” (the proportional distribution of these ASVs), and as an index combining elements of “Richness” and “Evenness” (the Shannon-Wiener index), using the *diversity* function of the *vegan* package of R (Oksanen et al. 2022). For modeling the error distribution in these GLMMs, an array of distributions were used: logit-link Bernoulli (prevalence), LogNormal (virus loads), Gamma (Shannon-Wiener and Evenness), and log-link Poisson (richness) distributions (see Supplementary Appendix 1). We also considered whether DWV genetic diversity measures were related to DWV loads. In these models, it was assumed that the identity of the ASV dominating the DWV quasi-species in each sample (the single most abundant ASV, or “dominant” ASV), as well as the extent of its dominance, would influence the different measures of genetic diversity. Therefore, it was necessary to include the identity of the dominant ASV in each sample as a random effect on the intercept and its proportional dominance in the sample’s quasi-species (i.e. its relative abundance among the sample’s DWV reads) as a fixed effect, in addition to DWV load (see Supplementary Appendix 1 for the formal model descriptions). Modelling was implemented in a Bayesian framework using JAGS (Plummer 2003) called from R (R Core Team 2022). A Bayesian approach was used because it allows probabilities of the direction and magnitude of effect sizes to be directly calculated. For all models, we used minimally informative priors and sampled the Monte Carlo Markov Chains for 10 000 iterations after chain convergence had been reached, as determined by visual inspection of stability and mixing. Posterior predictive checks were used to check model fit.

## Supplementary Material

veae053_Supp

## Data Availability

The DWV sequences generated are available in the GenBank (www.ncbi.nlm.nih.gov) Small Read Archives (SRA) under BioProject Number PRJNA1071107 and Accession Numbers SRX23449634–SRX23449533. The DWV-C most abundant sequences (curated) were also deposited in GenBank Nucleotide database under accession numbers PP259342–PP259351. The numerical data used in this study are found in the supplementary files, in Supplementary Tables S1 and S2.

## Figures and licensing

All figures were our own work except for the Azores base maps in Figs 3, 6, and Supplementary Fig. S4, and the varroa icon used in Figs 1 and 4. The Azores location and topographical maps in Fig. 3 and Supplementary Fig. S4 were constructed with the open-source geographic information system software QGis 3.22 (https://qgis.org) using a base map file downloaded from DIVA-GIS (www.diva-gis.org), both released under the GNU General Public License (GPL). The varroa icon was created at BioRender.com and was downloaded under a student/postdoc license, which permits use and publication in scientific journals. The maps used in Fig. 6 are freely available for academic use from www.pptmaps.com. The heatmap surrounding the phylogenetic tree in Fig. 2a was conducted in the online tool iTOL (https://itol.embl.de). The text and images created by the authors were superimposed on the maps for illustrative purposes.
