# Supplementary material for: Origins, diversity, and adaptive evolution of DWV in the honey bees of the Azores: the impact of the invasive mite Varroa destructor
Source: Virus Evol. 2024 Jul 15;10(1):veae053. doi: 10.1093/ve/veae053 (PMC11306321; doi:10.1093/ve/veae053)
Supplement: veae053_Supp [file veae053_supp.zip › veae053_Supp/suppl_data/Lopes2024-VirusEvol-Azores DWV evolution ecology APP_revised.docx]

**Supplementary Appendix 1**

These are the formal model descriptions for the Bayesian statistical modelling.

**1. Model relating DWV prevalence (as our response variable) to varroa presence.** Here DWV was determined to be present or absent in a colony sample. Thus we used a Bernoulli (i.e. a 1/0 binomial) distribution with a logit-link to estimate the importance of the varroa-status of the island the colony was located (varroa), and the sampling year (year). Because colonies were sometimes sampled from the same apiaries, we included ‘apiary’ as a random effect on the intercept to adjust for local factors that might influence the probability of DWV infection. The index ‘i’ indicates individual colonies sampled for DWV, and the index ‘j’ indicates the number of apiaries sampled. The Bernoulli parameter ‘p’ is the estimated prevalence of DWV

**Hierarchical model structure**

DWV presence_i_ ~ Bernoulli (p_i_)

logit(p_i_) = intercept _j_ + b1*varroa_i_ + b2*year_i_ **eq. (1)**

intercept _j_ ~ Normal (mu, sigma)

**Priors**

mu~Normal(0, 100)

sigma~Uniform(0,5)

b1~Normal(0,100)

b2~Normal(0,100)

**2. Model relating DWV load (log-transformed as our response variable) to varroa presence.** Here we restricted the dataset only to colonies that were positive for DWV (i.e. excluded all measures of zero from the analysis; thus this analysis is independent of the prevalence analysis in equation 1). DWV load was a highly-skewed continuous variable and the log-transformation of data normalised the response variable (thus making the analysis a LogNormal model). Here we used the same basic model structure as used above in equation 1, relating DWV load to varroa island status (varroa) and year of sampling (year), with apiary included as a random effect in the intercept. Because colonies were sometimes sampled from the same apiaries, we included ‘apiary’ as a random effect on the intercept to adjust for local factors that might influence the probability of DWV infection. The index ‘i’ indicates individual colony samples for DWV load, and the index ‘j’ indicates the number of apiaries sampled.

**Hierarchical model structure**

DWV load_i_ ~ Normal (mu_i,_ sigma)

mu_i_ = intercept _j_ + b1*varroa_i_ + b2*year_i_ **eq. (2)**

intercept_j_ ~ Normal (mu2, sigma2)

**Priors**

mu~Normal(0, 100)

sigma~Uniform(0,5)

mu2~Normal(0, 100)

sigma2~Uniform(0,5)

b1~Normal(0,100)

b2~Normal(0,100)

**3. Models relating measures of DWV diversity (i.e. Richness, Evenness and Shannon-Wiener index) to varroa presence.** Here we restricted the dataset only to colonies that were positive for DWV (i.e. excluded all measures of zero from the analysis). Diversity measures varied in that richness was a count of DWV variants, and thus we used a Poisson distribution to describe them (with a log-link), while evenness and Shannon-Wiener were positive continuous data and so we used a Gamma distribution to describe these (with an identity-link). Again we used the same basic model structure as used above in equation 1, relating DWV diversity to varroa island status (varroa), with apiary included as a random effect in the intercept. The index ‘i’ indicates individual colony samples for DWV load, and the index ‘j’ indicates the number of apiaries sampled.

**Hierarchical model structure**

**Richness**

DWV richness_i_ ~ Poisson (lambda_i_)

log(lambda_i_) = intercept_j_ + b1*varroa_i_ **eq. (3)**

intercept_j_ ~ Normal (mu, sigma)

**Evenness / Shannon-Wiener**

DWV evenness_i_ ~ Gamma (a_i,_ b_i_) **eqs (4 & 5)**

DWV Shannon_i_ ~ Gamma (a_i,_ b_i_)

mu_i_ = intercept_j_ + b1*varroa_i_

intercept _j_ ~ Normal (mu2, sigma2)

a_i_ = mu_i_ ^2 / sigma^2

b_i_ = mu_i_ / sigma^2

**Priors for eqs 3,4 & 5**

mu~Normal(0, 100)

mu2~Normal(0, 100)

sigma~Uniform(0,5)

sigma2~Uniform(0,5)

b1~Normal(0,100)

**4. Models relating measures of DWV diversity (i.e. richness, evenness and Shannon-Wiener index) to DWV loads.** Here we restricted the dataset only to colonies that were positive for DWV (i.e. excluded all measures of zero from the analysis). Diversity measures varied, in that richness was a count of DWV variants, and thus we used a Poisson distribution to describe them (with a log-link), while evenness and Shannon-Wiener were positive continuous data and so we used a Gamma distribution to describe these (with an identity-link). With these models we considered the potential for competition between different DWV-variants, such that the dominant strain might influence the overall diversity in a colony (either in a positive or negative way). Thus, for these models to account for this potential influence of the dominant strain, we used the identity of the dominant strain as a random effect on the model intercept to capture this effect. We also considered that the degree of ‘dominance’ of the dominant DWV-variant might negatively influence diversity, so we included proportion of reads that found the dominant variant (prop.dominant.ASV) as an additional explanatory factor in the model, alongside the DWV-load which is what we were primarily interested in. The index ‘i’ indicates individual colony samples for DWV diversity measures, DWV titre & proportion-reads of the dominant strain, and the index ‘j’ indicates the the DWV variant that was dominant in the colony sampled.

**Hierarchical model structure**

**Richness**

DWV richness_i_ ~ Poisson (lambda_i_) **eq. (6)**

log(lambda_i_) = intercept_j_ + b1*DWV load_i_+ b2*prop.dominant.ASV

intercept_j_ ~ Normal (mu, sigma)

**Evenness / Shannon-Wiener**

DWV evenness_i_ ~ Gamma (a_i,_ b_i_) **eqs (7 & 8)**

DWV Shannon_i_ ~ Gamma (a_i,_ b_i_)

mu _i_ = intercept_j_ + b1*DWV load_i_ + b2*prop.dominant.ASV

intercept_j_ ~ Normal (mu2, sigma2)

a_i_ = mu_i_ ^2 / sigma^2

b_i_ = mu_i_ / sigma^2

**Priors eqs 6,5 & 7**

mu~Normal(0, 100)

mu2~Normal(0, 100)

sigma~Uniform(0,5)

sigma2~Uniform(0,5)

b1~Normal(0,100)
