# Supplementary material for: Origins, diversity, and adaptive evolution of DWV in the honey bees of the Azores: the impact of the invasive mite Varroa destructor
Source: Virus Evol. 2024 Jul 15;10(1):veae053. doi: 10.1093/ve/veae053 (PMC11306321; doi:10.1093/ve/veae053)
Supplement: veae053_Supp [file veae053_supp.zip › veae053_Supp/suppl_data/Lopes2024-VirusEvol-Azores_DWV_evolution_ecology_SUPFIG_CLEANpdf.pdf]

Supplementary Figures

Origins, diversity, and adaptive evolution of DWV in the honey bees of the Azores: the impact of the invasive mite *Varroa destructor*

Ana R. Lopes[1,2,3], Matthew Low[4], Raquel Martín-Hernández[5,6], M. Alice Pinto[1,2#*], Joachim R. de Miranda[4#*]

[1]Centro de Investigação de Montanha (CIMO), Instituto Politécnico de Bragança, Campus de Santa Apolónia, 5300-253 Bragança, Portugal

[2]Laboratório Associado para a Sustentabilidade e Tecnologia em Regiões de Montanha (SusTEC), Instituto Politécnico de Bragança, Campus de Santa Apolónia, 5300-253 Bragança, Portugal

[3]REQUIMTE-LAQV, Faculdade de Farmácia, Universidade do Porto, Rua de Jorge Viterbo Ferreira, 228, 4050-313 Porto, Portugal

[4]Department of Ecology, Swedish University of Agricultural Sciences, 750-07 Uppsala, Sweden

[5]Centro de Investigación Apícola y Agroambiental (CIAPA). IRIAF. Instituto Regional de Investigación y Desarrollo Agroalimentario y Forestal, 19180 Marchamalo, Spain

[6]Instituto de Recursos Humanos para la Ciencia y la Tecnología (INCRECYT-FEDER), Fundación Parque Científico y Tecnológico de Castilla—La Mancha, 02006 Albacete, Spain

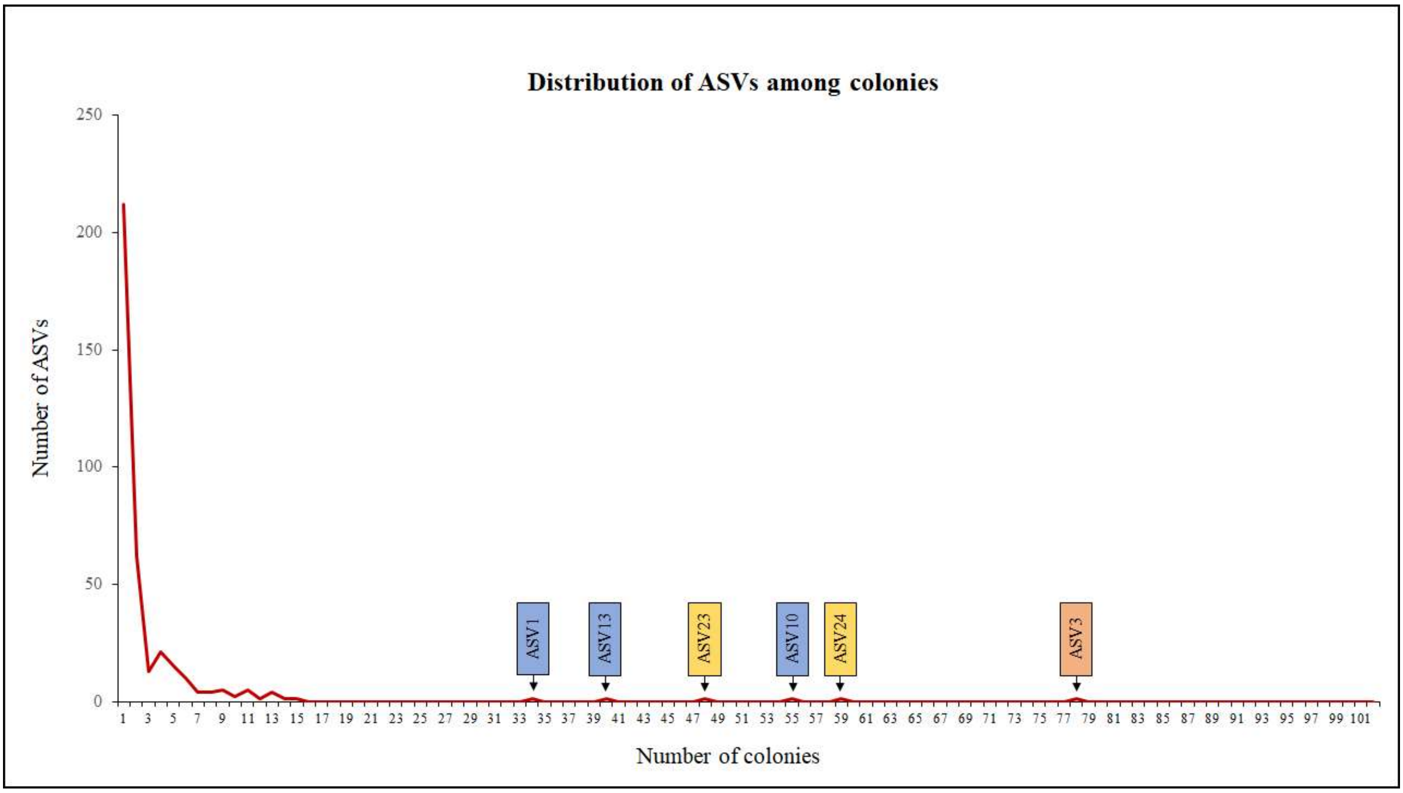

Fig. S1. The distribution of the 366 DWV ASVs among the 102 colony samples. The graph shows the number of ASVs that are detected in only one, two, three *etc.* different colonies. Also shown are the identities and clades of the six most common ASVs, found in large numbers of colonies. Blue ASVs belong to Clade-A, yellow ASVs to Clade B and orange ASVs to Clade-C.

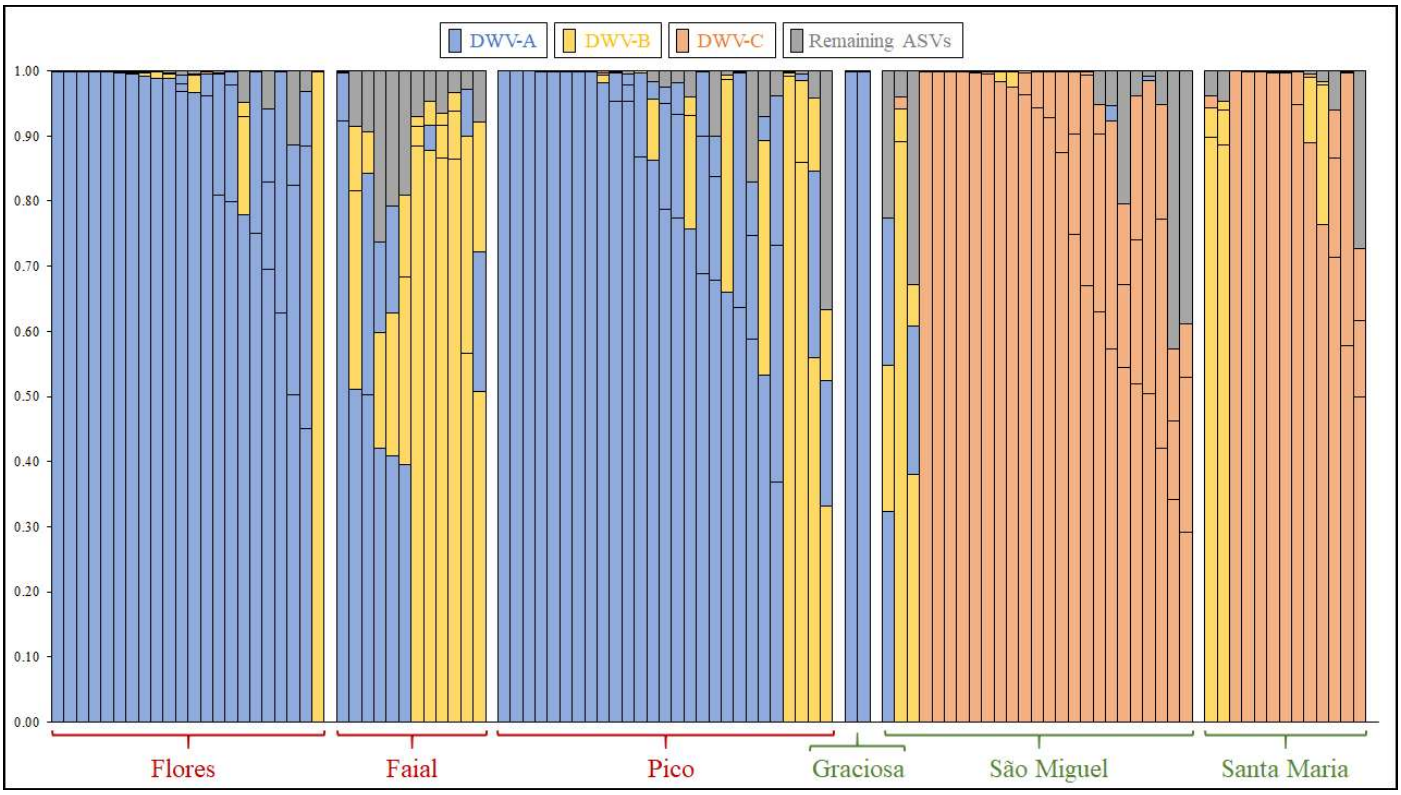

Fig. S2. The proportions and clades of the top three most abundant ASVs in each DWV quasi-species, separated by the six islands. The proportion of all remaining ASVs combined are represented by a grey bar.

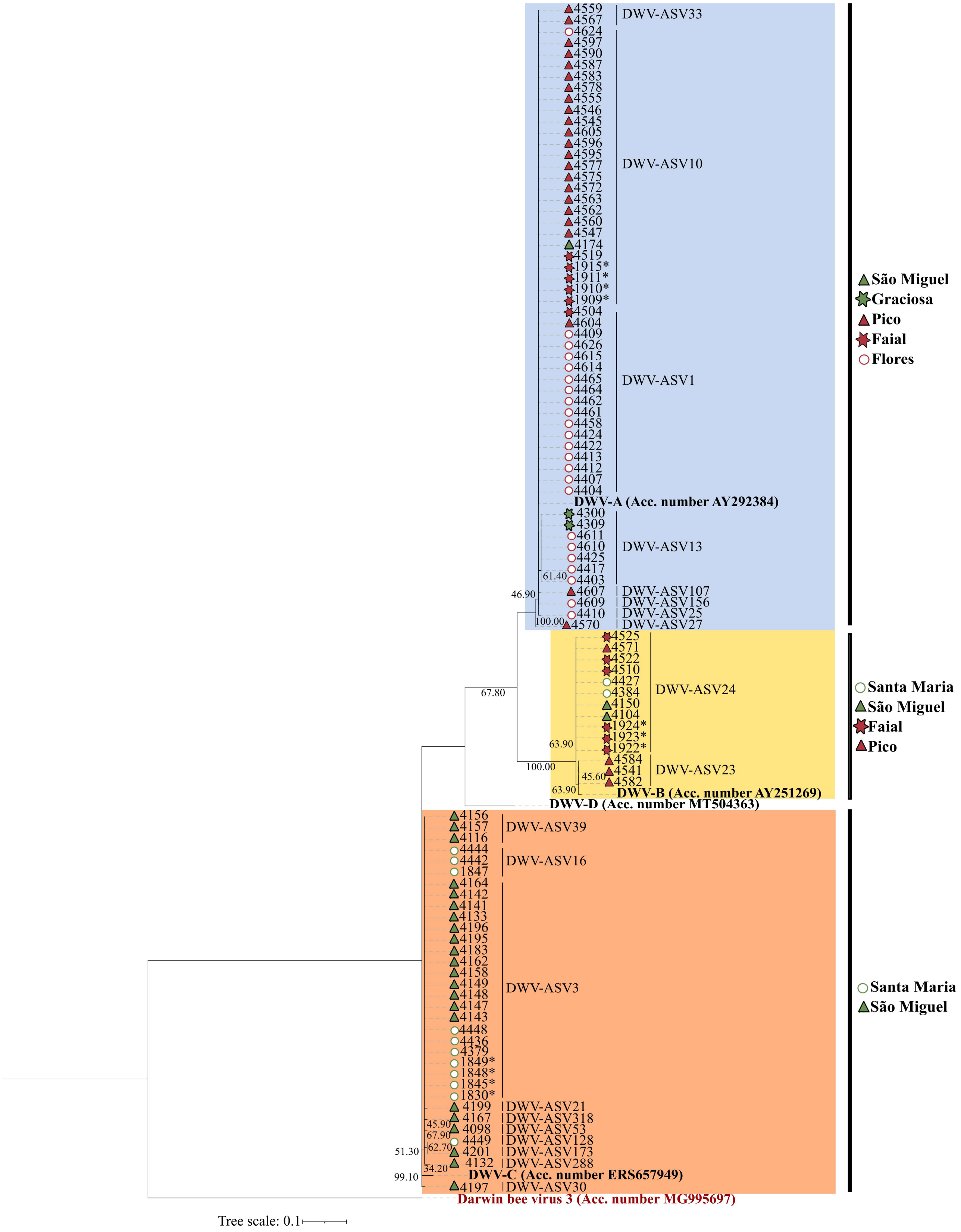

Fig. S3. Phylogeny and distribution of the 20 single most abundant 'dominant' DWV ASVs identified in the 102 DWV-positive colony samples. DWV-master references from GenBank are marked in bold. Darwin bee virus 3 (red) was selected as the phylogenetic outgroup. The evolutionary history was inferred from the maximum likelihood method using the Tamura 3-parameter model (bootstrap = 1000 replicates). The green icons denote the islands without varroa and red-icons the islands with varroa. Colonies sampled in 2020 are marked with a star sign (*).

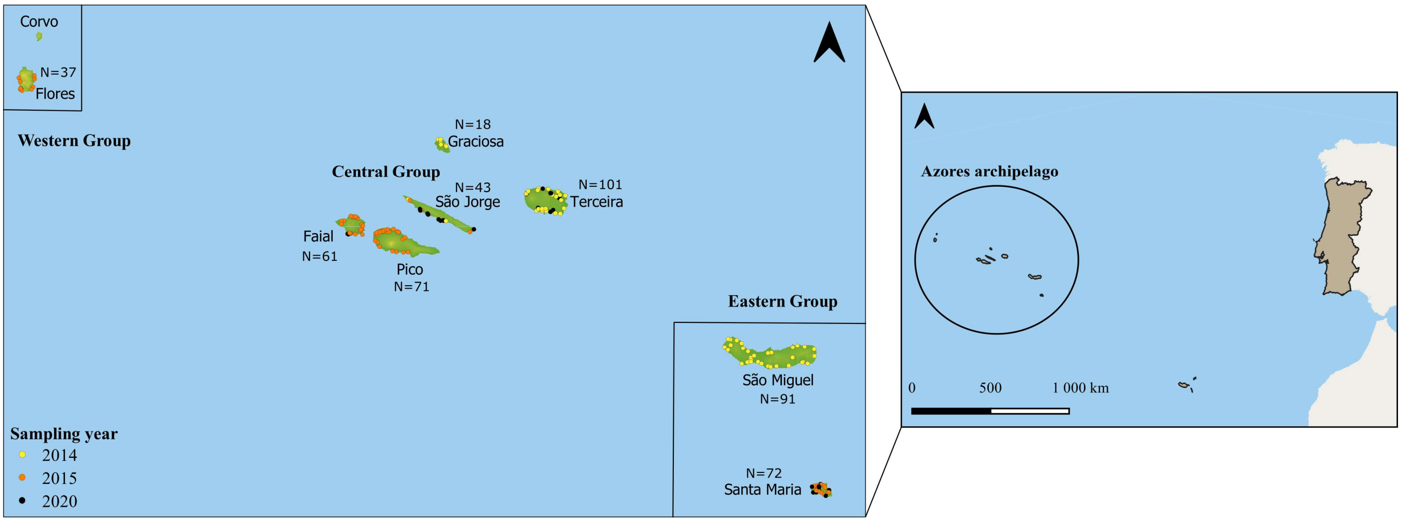

Fig. S4. Geographic distribution of sampled apiaries in the Azores by sampling year. N is the number of sampled colonies.
